# Optimized Neural Network Based on Genetic Algorithm to Construct Hand-Foot-and-Mouth Disease Prediction and Early-Warning Model

**DOI:** 10.3390/ijerph18062959

**Published:** 2021-03-14

**Authors:** Xialv Lin, Xiaofeng Wang, Yuhan Wang, Xuejie Du, Lizhu Jin, Ming Wan, Hui Ge, Xu Yang

**Affiliations:** 1 School of Computer Science and Technology, Beijing Institute of Technology, Beijing 100081, China; 3220201066@bit.edu.cn (X.L.); 3220180872@bit.edu.cn (Y.W.); 2 Chinese Center for Disease Control and Prevention, Beijing 102206, China; wangxf@chinacdc.cn (X.W.); duxj@chinacdc.cn (X.D.); jinlz@chinacdc.cn (L.J.)

**Keywords:** hand-foot-and-mouth disease, early-warning model, neural network, genetic algorithm

## Abstract

Accompanied by the rapid economic and social development, there is a phenomenon of the crazy spread of many infectious diseases. It has brought the rapid growth of the number of people infected with hand-foot-and-mouth disease (HFMD), and children, especially infants and young children’s health is at great risk. So it is very important to predict the number of HFMD infections and realize the regional early-warning of HFMD based on big data. However, in the current field of infectious diseases, the research on the prevalence of HFMD mainly predicts the number of future cases based on the number of historical cases in various places, and the influence of many related factors that affect the prevalence of HFMD is ignored. The current early-warning research of HFMD mainly uses direct case report, which uses statistical methods in time and space to have early-warnings of outbreaks separately. It leads to a high error rate and low confidence in the early-warning results. This paper uses machine learning methods to establish a HFMD epidemic prediction model and explore constructing a variety of early-warning models. By comparison of experimental results, we finally verify that the HFMD prediction algorithm proposed in this paper has higher accuracy. At the same time, the early-warning algorithm based on the comparison of threshold has good results.

## 1. Introduction

With the intensification of global warming, climate abnormalities and natural disasters have become more and more intense, and the increasing changes in the environment have provided very favorable conditions for the spread of hand-foot-and-mouth disease (HFMD) [1,2]. Although HFMD is not a critical disease, there are still many children who have very serious complications due to this illness. If they are not treated in time, a series of complications such as myocarditis and encephalitis will occur, causing vital organ damage and even threatening their lives [3].

The prevalence of HFMD in China has continued unabated, and it has received great attention from the national health department. The prevention and treatment of HFMD should stop transmission from the root cause. However, the virus that leads to HFMD is not only many kinds, but also many types. Therefore, to carry out research on the prediction of the number of HFMD prevalence, the early-warning of epidemic trends and related factors has become the top priority of the country’s HFMD epidemic control [4].

However, in previous studies on HFMD prediction and early-warning models, relevant researchers mainly conducted statistical analysis on factors related to the HFMD epidemic [5]. Including weather and demographic attributes, they explore the correlation between the different regions in the incidence of HFMD at different times of the amount of each factor and established a variety of models for prediction. However, these methods lack accurate positioning and research on the HFMD epidemic warning, and the data for establishing the prediction model is insufficient, the time and space of the data are too large, and the method used by the model is not perfect. Above shortcomings have caused many problems such as HFMD prediction and early-warning model to be inaccurate, limited to the problems of broad prediction and blind early-warning.

This paper aims to carry out accurate data analysis and standard data preprocessing based on the incidence of HFMD. At the same time, this paper also established a more accurate prediction model of the number of HFMD cases and a more reasonable early warning model. These have laid the foundation for realizing early warning of whether the regional HFMD has broken out or strengthened prevention and control.

## 2. Related Work

The World Health Organization (WHO) attaches great importance to the establishment of an early-warning system for infectious diseases, and develops an early-warning mechanism for infectious diseases and promoted its irreplaceable important role [6]. The principle of the early warning system is to make a judgment on whether there will be an outbreak or epidemic of infectious diseases based on the clinical information of the existing disease diagnosis patients. Their purpose is clear, just to detect abnormal health incidents promptly, quickly notify relevant health departments and staff, and take preventive and control measures in the first time. The early-warning system of such infectious diseases is the symptom monitoring system [7,8].

Since its establishment in 1946, the Centers for Disease Control and Prevention (CDC) has established a national infectious disease surveillance system for epidemic infectious diseases such as malaria and influenza. Until 1995, they began to build for all types of acute infectious disease monitoring network, in 2001 they integrated more than 100 spotty infectious disease surveillance system. Since then, the monitoring and early-warning system has been changed to “National Disease Electronic Monitoring System” [9].

The European Union (EU) has developed a group-type infectious disease monitoring and early-warning system based on the cooperation of all member states. It provides a collaborative platform for information and control and prevention for the countries in the group, and at the same time focuses on international cooperation and exchanges [10].

In January 2004, China began trial operation of the direct online reporting system for epidemics and public health emergencies, and the system was officially launched in April of the same year [11]. Afterwards, direct online reporting of various infectious diseases such as tuberculosis, dengue fever, and HFMD have been launched on the system, and public health information resources have been integrated and shared. At present, China’s infectious disease early-warning model mainly uses the mobile percentile early-warning method and the spatial scanning statistical method [12,13]. However, these two methods rely too much on the direct reporting system of infectious diseases, and because the model is simple, many parameters are determined artificially. This has led to the problems of poor early-warning accuracy, repeated early-warnings, no early-warnings during epidemic periods and chaotic early-warning during non-epidemic periods, which seriously affected the early-warning work of HFMD epidemics.

At present, most researchers’ research on the prevalence of HFMD relies on statistical methods such as multiple linear regression [14], cross-correlation analysis, and correlation analysis. They analyzed the correlation between related influencing factors and the incidence of HFMD, and obtained statistically significant results. This research results mostly proved the correlation between certain epidemic factors and the number of HFMD cases; at the same time, the incidence of HFMD epidemic was predicted by using the above-mentioned three infectious disease prediction methods.

Yin Ye et al. counted the daily incidence of HFMD for six years since 2011, calculated the correlation coefficient between the daily incidence of HFMD and twelve weather indicators of the day, and drew the conclusion that the daily incidence of HFMD is correlated with certain meteorological factors [15]. Jing Qinlong et al. used cross-correlation analysis methods to study relevant meteorological factors. They found that as the lag period decreases, the relationship between monthly average temperature and monthly cumulative precipitation and the number of HFMD monthly cases is the strongest [16]. Liu Yamin et al. established a variety of different prediction models using monthly incidence data from 2010 to 2015, then input the monthly incidence rate data of HFMD in 2016 as test data into the model [17]. Under the comparison of four objective evaluation indicators, they found that the seasonal autoregressive integrated moving average (SARIMA) model not only has excellent fitting generalization ability, but also has higher prediction accuracy.

As we entered the era of big data, many scholars began to design methods using big data to help build more accurate prediction or early-warning model of diseases [18,19]. So in this paper, we would present our effort at constructing a HFMD prediction and early-warning model with the help of big data.

## 3. Construct HFMD Prediction Algorithm Model Based on BP Neural Network

**Figure 1:**
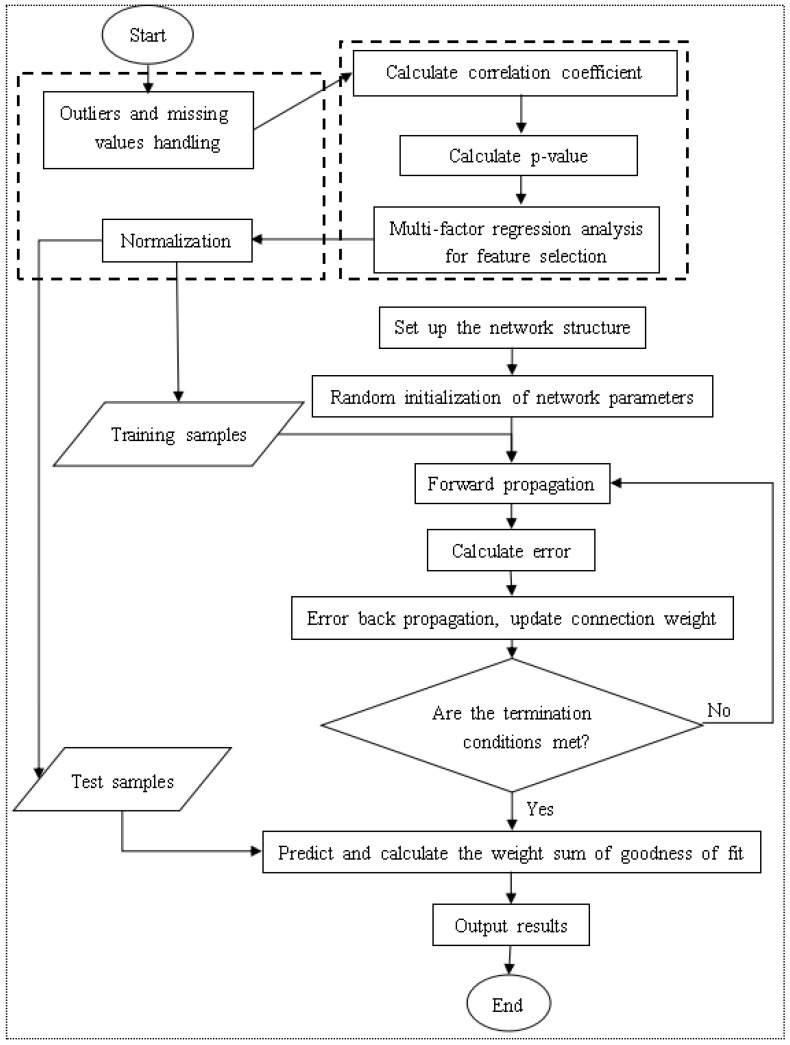
Flow chart of HFMD epidemic prediction model based on BP neural network.

Figure 1 shows the overall process of the HFMD prevalence prediction model based on back propagation (BP) neural network constructed in this article, which will be introduced in detail below.

### 3.1. Data Acquisition and Analysis

This paper uses big data to build a predictive and early-warning model for HFMD through multi-dimensional data fusion. The data used mainly include two parts: incidence data and environmental data.

First of all, the incidence data comes from HFMD in Shanxi Province in 2016. There is no personal privacy data in this data, including: region (township), date of onset, age group, gender group, and population classification.

**Figure 2:**
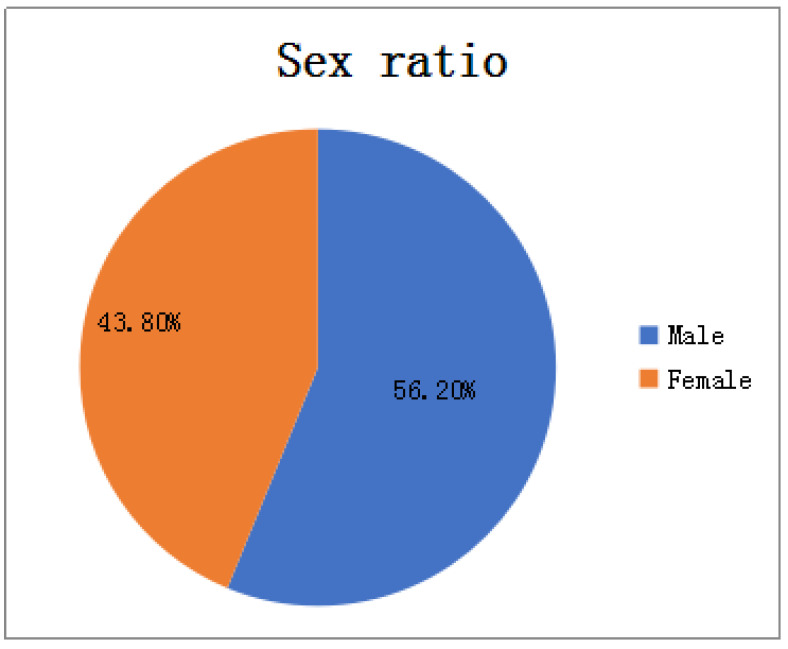
Sex ratio of HFMD patients.

**Figure 3:**
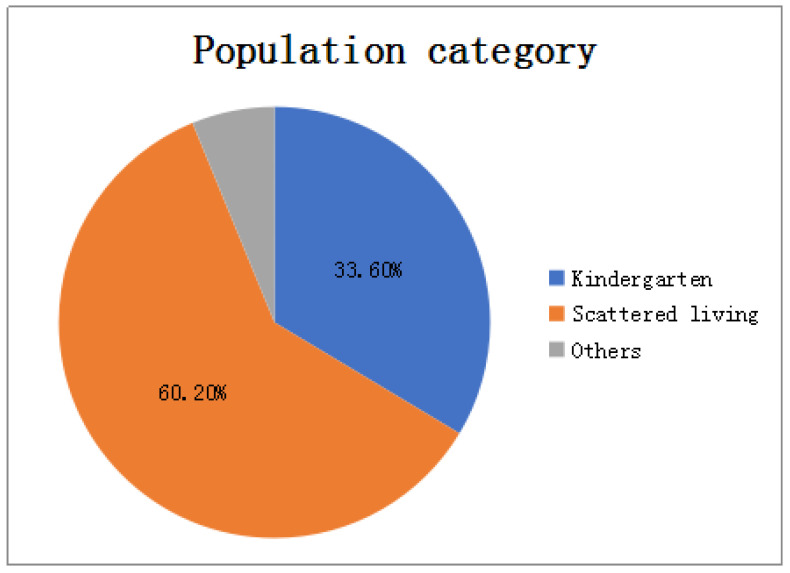
HFMD’s proportion of each population category.

**Figure 4:**
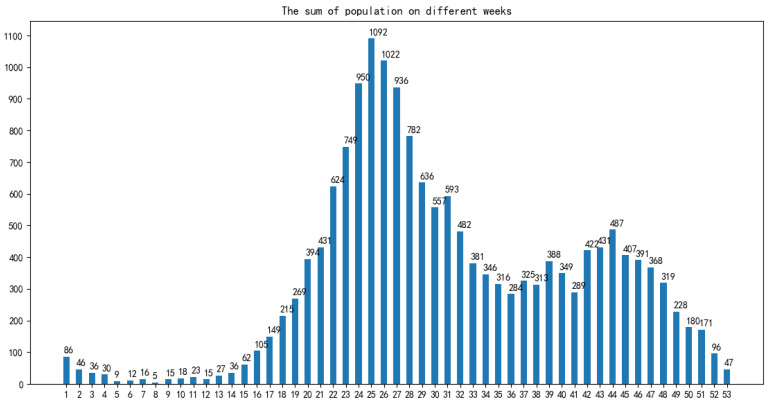
HFMD’s proportion of each population category.

**Figure 5:**
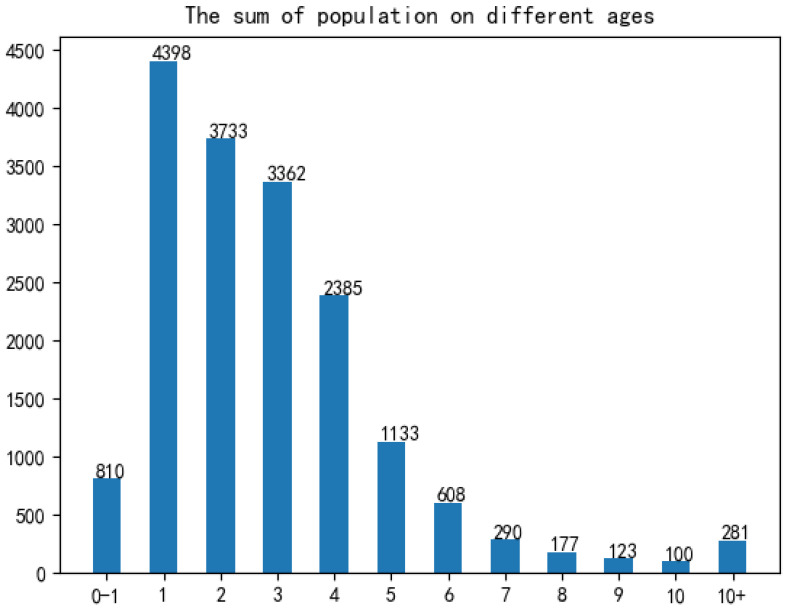
Age distribution of HFMD epidemic. the link (http://data.sheshiyuanyi.com/WeatherData/, accessed on 1 March 2020). x-axis is in years.

For the incidence data, we carried out exploratory data analysis to select appropriate characteristic factors affecting the HFMD epidemic in the model construction process, mainly analyzing indicators such as gender, population type, onset time, and patient age:

1. In terms of gender: As shown in Figure 2, among all HFMD patients in the province in 2016, the ratio of male to female patients was about 4:3. It can be considered that the relationship between HFMD infection and gender is very small, i.e., the chances of male and female being infected with HFMD are equal, so the ratio of men to women is not considered as a relevant factor affecting the prevalence of HFMD.
2. In terms of population types: As shown in Figure 3, there are three types of populations for all patients: kindergarten, scattered living, and other categories. The proportions of patients are 33.6%, 60.2%, and 6.2% respectively. Patients infected with HFMD are mainly concentrated in kindergartens and scattered populations, but the proportion of scattered patients is twice that of kindergarten patients, so the number of children in kindergartens cannot be a good predictor of HFMD infection patients.
3. In terms of time: As shown in Figure 4, the infection time of patients is mainly concentrated in 22–32 weeks (June-August). The 24th, 25th, and 26th week is the HFMD epidemic period, and the number of infections reaches a large peak. There are also multiple occurrences in 36–48 weeks, reaching a small peak of infection around the 44th week. Therefore, the prevalence of HFMD is characterized by a strong seasonal infection. The relevant weather indicators can be used as one of the important factors in determining the prevalence of HFMD.
4. In term of age: As shown in Figure 5, infants and children aged 0–6 years of HFMD infection account for a considerable portion, accounting for about 95% of all infected people.Therefore, the number of children aged 0–6 in each region can be used as an important indicator to predict the prevalence of HFMD.

After the epidemic analysis of the original data, according to the number of cases per day in each district and county, the statistics are summarized, and only the date, area and statistical incidence in the original data file are retained.

Then, according to the results of epidemic analysis, the daily weather of each district and county was obtained. Due to different weather data sources and different ways of data acquisition, some weather data (maximum temperature, minimum temperature, wind level) need to be obtained from lishi.tianqi.com by web crawler; the other part of weather data (sunshine duration, air humidity, average air pressure) is obtained by file download. Because this part of the weather data only exists in the meteorological stations in the province and the data index is stable, the three weather data of 18 meteorological stations in Shanxi Province are downloaded from the http://data.sheshiyuanyi.com/WeatherData/, accessed on 1 March 2020, and the statistical areas are allocated according to the weather data of the nearest meteorological stations. Before the distribution, the nearest meteorological stations can be found by crawling the geographical location of the regions and meteorological stations, i.e., latitude and longitude data, and the three weather data of the nearest stations are allocated to the statistical areas; to consider the effect of the incubation period (usually 4 days) on the daily incidence of disease, the corresponding weather data of the day before 4 days were obtained, and the weather indexes such as maximum temperature, minimum temperature, wind grade, average sunshine duration, average air humidity and average air pressure were obtained in the same way.

Finally, according to the results of HFMD epidemic analysis, population data needs to be summarized, so the internal population data is calculated to count the number of children aged 0–6 in each region, and integrated into the data file generated in the previous step; at the same time, in order to consider the impact of the incidence of the day before the day on the day, the number of cases from the previous day is also included in the model characteristics to generate complete data for establishing the HFMD epidemic prediction model.

### 3.2. Data Preprocessing

The process of data preprocessing will greatly influence the result of data analysis [20].

#### 3.2.1. Missing Value Processing

Among the data related to the factors affecting the spread of HFMD, the weather data or the population data of districts and counties on the day have some variable values missing, so appropriate methods must be used to deal with them. First of all, for variables whose values are not collected and most of the individuals whose variables are missing, the simple deletion method is used to directly delete variables or individual data, and will not be included in experimental research and data analysis. Then, the nearest neighbor padding method is used to fill the attributes with stable attribute values and small numerical variance. Finally, the mean value filling method is used to deal with the situation where a small part of the data is missing.

#### 3.2.2. Outlier Handling

The regional weather data obtained by the web crawler is identified through outliers, and it is found that some data is abnormal, so the outliers need to be replaced or corrected. First, for univariate factors, define constraints that meet actual needs, and use the mean replacement method for variable outliers that do not meet the constraint definition. That is, when the value of a certain variable of a certain object is found to be abnormal, the average value of all other normal and non-missing values on the variable is calculated to replace the abnormal value. Secondly, for multiple variable factors, the order of the highest temperature and the lowest temperature often changes due to changes in the structure and content of the crawler page. Therefore, it is necessary to identify the individual data with the lowest temperature higher than the highest temperature, and exchange the two to make the data meet the constraints.

#### 3.2.3. Data Standardization

The Z-score standardization method used in this paper. This paper also uses a simple downgrading standardization method, because the number of population plays a crucial role in the incidence of HFMD, and the magnitude of the difference between the number of population and the actual incidence of HFMD is large, which is not conducive to analysis. Therefore, the value of this variable is degraded.

### 3.3. Feature Selection

Through the data preprocessing process, we finally established the data table shown in Table 1.

When constructing the supervised learning model for the prediction of the number of HFMD cases, we maximized the fact that many a priori unknown related features (meteorological and demographic features) were incorporated into the learning objectives. So that the target problem (the number of HFMD cases) can be trained and learned more effectively. However, some of the related features are not very relevant to the learning goal, or even have no relationship. These features are usually called redundant features. When they are added to the learning task, problems such as poor learner performance and data disaster are likely to occur. Therefore, it is very necessary to select all features to greatly enhance the generalization ability of the prediction model. This paper uses a multivariate joint feature selection method based on correlation analysis.

In the study of the HFMD epidemic prediction model, three comprehensive feature selection algorithms including filtering, wrapping and embedding are used. Different methods are used in different training and learning stages, using filtering algorithms before training, using embedded algorithms during training, and using wrapped algorithms after training. In this way, the feature subset with the best performance is selected, the learner with the strongest generalization ability is selected, and the number of cases is predicted more accurately for scientific prevention and control.

The core of the embedded algorithm is to integrate the feature selection process into the model learning process, and the features are selected cleverly while learning, so the algorithm depends on the machine learning algorithm used. However, embedded algorithms are not used in the preprocessing of data in the early stage, and only used during model training.

The core of the wrapped algorithm is to directly use the evaluation index of the learner to reflect the pros and cons of the feature subset. The higher the accuracy of the learner, the better the feature subset. Therefore, it is necessary to repeatedly use different feature subsets to construct multiple learners until the best learner is obtained and the best feature subset is obtained.

The core of the filtering algorithm is to directly filter out undesirable features to filter out relatively good feature subsets. Then, without training the model, use an appropriate evaluation function to evaluate the pros and cons of the feature subset until the best evaluated feature subset is selected. Therefore, the feature selection of this method is independent of the target learner, and the advantage is that it is simple, efficient and fast.

Before the actual training of the learner, the filter method is usually used to select the features, and the dependency metric is used to evaluate the feature subset. At the same time, according to the results of the dependency measurement, the measurement threshold is set, and the features whose relevant indicators are greater than the threshold are selected, and further statistically significant tests are performed on them as a double standard for selecting features. At the same time, bivariate correlation analysis is difficult to escape the influence of confounding factors, so multiple linear regression analysis methods must be used to establish a regression model for the influencing factors and the number of hand, foot and mouth cases, and find the secondary confounding factors according to the partial regression coefficients. The previous filtering feature selection process is completed.

The selection process can be divided into the following steps:

1. Calculate the variance of all variables using a single variable analysis method;
2. Filter out attributes whose variance is greater than the variance threshold, and get a preliminary feature subset;
3. Using bivariate correlation analysis method, calculate the Pearson coefficient, Spearman coefficient, distance correlation coefficient and *p*-value of the independent variable and the dependent variable;
4. According to the correlation coefficient and statistical *p*-value results, select the features whose *p*-value is less than the significance level and the correlation coefficient is greater than the coefficient threshold to obtain a more accurate initial feature subset;
5. According to the feature subset selected by the variable analysis method, establish a multivariate joint regression model based on the multiple linear regression model, and obtain the partial regression coefficient, intercept and statistical *p*-value of the model;
6. According to the multiple regression parameter table, filter and select variables whose *p* value is less than the significance level, and obtain the feature subset in the linear model;
7. Then the k features with the largest nonlinear correlation coefficients in the initial feature subset, which do not exist in the linear model feature subset, are included in the nonlinear model feature subset.

### 3.4. Construction of HFMD Prediction Model

Commonly used machine learning regression prediction algorithms include multiple linear regression (LR), support vector regression (SVR), differential integrated moving average autoregressive models and BP neural networks [15] and so on. Through analysis, we will select BP neural network to construct an early prediction model for HFMD on big data.

After analysis of related factors, seven related variables were obtained. After these seven related variables are normalized by Min-Max, the training set and the test set are randomly selected according to the ratio of 7:3. Then input the training set into the prediction model to be established, and train and adjust the parameters in the model. After a series of training processes, a HFMD epidemic prediction model suitable for solving this problem is obtained. Then input the test set into the HFMD prevalence prediction model for prediction, obtain the prediction result, and compare the result with the expected output to evaluate the HFMD prevalence prediction model.

**Figure 6:**
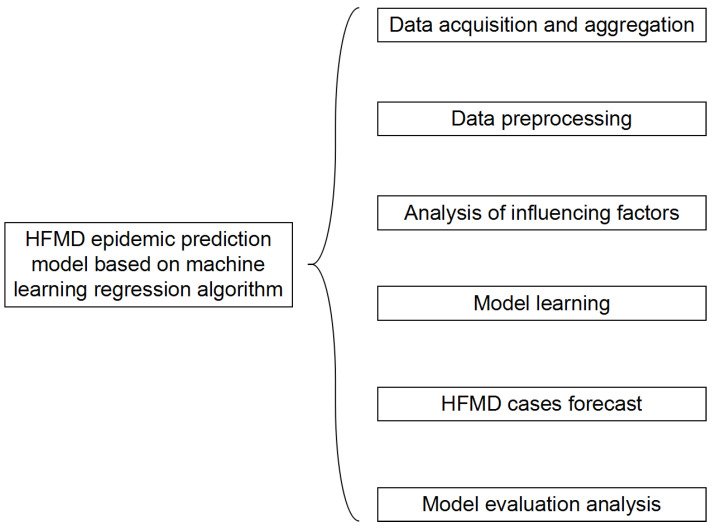
Structure diagram of HFMD epidemic prediction model based on machine learning regression algorithm.

The structure of the HFMD prediction model based on the machine learning regression algorithm is shown in Figure 6. It includes six modules: data acquisition and summary, data preprocessing, influencing factor analysis, model learning, epidemic case number prediction, and model evaluation analysis. In the data acquisition and summary module, the meteorological factors and demographic factors data related to the HFMD epidemic are acquired in multiple ways, and the county daily data is summarized as city weekly data; the dirty data is mainly cleaned in the data preprocessing module; in the influencing factor analysis module, univariate, bivariate and multivariate joint analysis of the correlation between influencing factors and the number of popular populations are carried out, and the feature set of relevant HFMD epidemic influencing factors suitable for modeling is selected; in the process of model learning, the machine learning regression model is used to learn to obtain the optimal structure; in the HFMD epidemic case number prediction module, the test set is input into the model; in the model evaluation and analysis module, the learned optimal model is analyzed with different weights on the training set and the test set, and the relevant evaluation index values are obtained to judge the pros and cons of the model.

**Figure 7:**
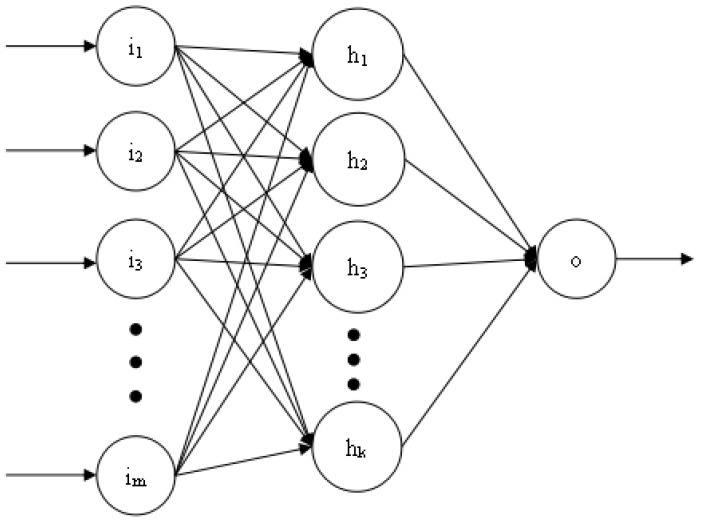
Three-layer single output BP neural network structure diagram.

Figure 7 shows the three-layer structure of the BP neural network used in this article. The number of neurons in each layer is *m*, *k*, and 1, respectively. The number of hidden layers and the number of neurons in each layer can be dynamically adjusted according to the training effect. However, the number of neurons in the first and last layers is fixed. The training process of BP neural network is realized by error feedback mechanism [16]. The activation function used is the relu function.

The model training process is:

1. Establish the network structure according to the actual HFMD prediction problem. There is only one input layer and output layer, which contain the number of neurons as the feature number and 1, respectively. A hidden layer with k neurons is initially set. If the training result is not Ideally, the number of layers and the number of neurons on each layer can be dynamically changed, but not more than three hidden layers;
2. Initialize the hyperparameters in the network structure, including learning rate, training times, and connection weights. If the training results are not ideal, the hyperparameter values can also be dynamically adjusted;
3. Start to input training samples into the network, obtain the predicted value of each sample through the forward propagation process, and calculate the overall error between the output predicted value and the expected value;
4. If the error does not meet the condition or the training does not reach the number of generations, the error is propagated back to the input layer, and the connection weight is updated in the process;
5. If it is greater than the set number of generations, the training process is ended, the structure of the BP neural network is output, and the test data is evaluated according to relevant indicators;
6. If the test result does not reach a certain threshold, it is necessary to adjust the relevant hyperparameters or the number of hidden layers or the number of neurons in each hidden layer in the network, and repeat the above training process.

## 4. Neural Network Parameter Optimization Based on GA

In the training process of BP neural network, the gradient descent method and error feedback propagation mechanism are essentially used to dynamically update the connection weights, which also exposes the shortcomings of this training method [21]. First of all, there are strict requirements for model hyperparameters such as learning rate, too large or too small learning rate will affect the optimization effect; Secondly, if the number of training iterations is too large, the convergence efficiency is low when the error function gradually becomes flat in the later stage, and it is difficult to converge to a flat point if the number of generations is too small; Finally, it is because the training starts according to the initial set of random weights, looking for a smooth gradient and falling into a local minimum state, it is difficult to jump out to find the global minimum state. Therefore, in view of the above shortcomings, we use genetic algorithm (GA) to globally optimize the connection weights.

First determine the BP network structure, and encode the target individual with floating-point numbers. Arrange all the connection weights in the neural network in order to form the row vector $W_{j}=\left(w_{1},w_{2},\cdots ,w_{n}\right)$ of individual *j*, which represents the genetic code of chromosome *j* in the population, The weight $w_{i}$ of connection *i* in the network represents the genotype of gene *i* on the chromosome, and *n* represents the number of all connections in the neural network. In this model, if the number of neurons contained in the input layer, the first hidden layer, the second hidden layer, and the output layer are *m, k, h,* 1, respectively, then the number of genes *n* is calculated as in Equation (1).

(1) n=m×k+k×h+h

Secondly, all individuals in the population must be initialized randomly. Each individual has a chromosome vector, which can be decoded back into a BP neural network model with floating-point numbers. Therefore, before learning, all individual vectors must be initialized with random real numbers in the range of [−1, 1] to generate the first generation population.

**Figure 8:**
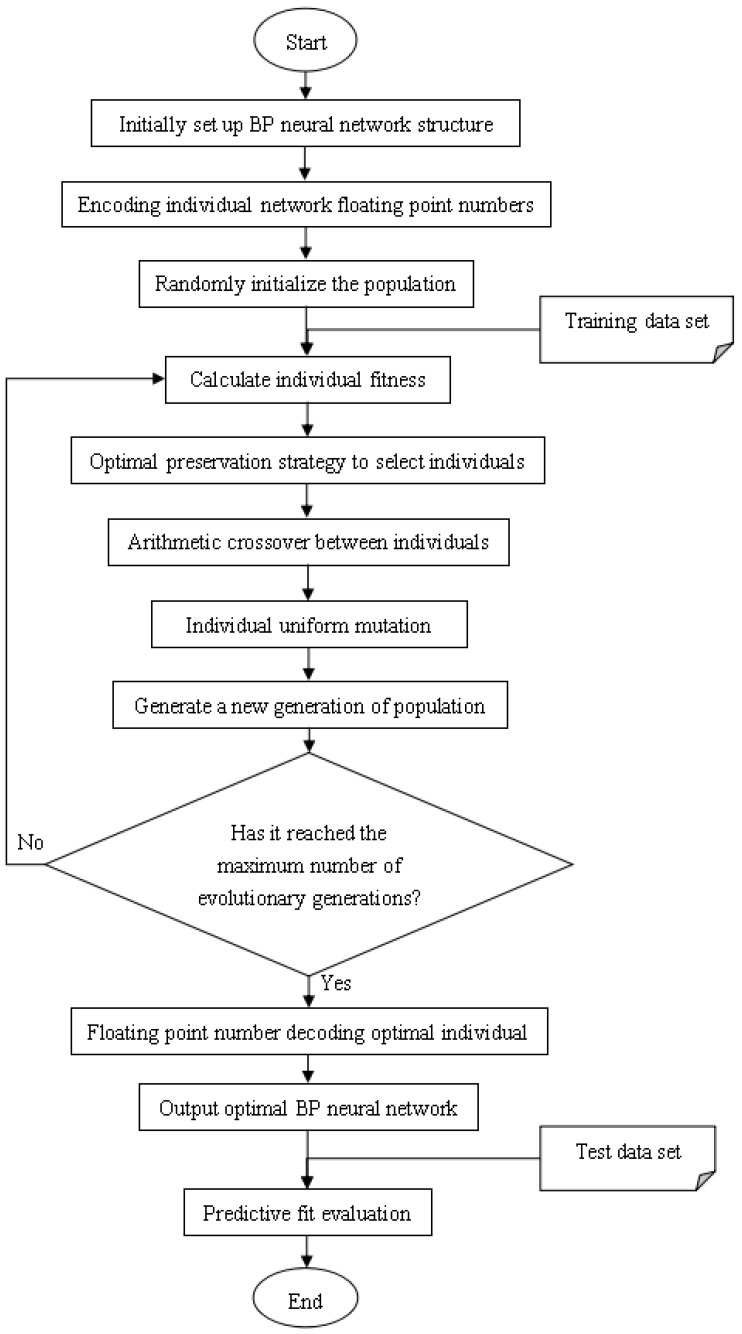
Flow chart of GA-BP HFMD epidemic prediction model.

Finally, it is necessary to calculate the fitness of all individuals, select individuals for genetic operations, including replication, crossover and mutation, to generate a new generation of populations [22]. In this paper, the fitness can be calculated directly from the average error of the individual on the sample. Therefore, select high fitness, i.e., individuals with small errors for retention, and select low fitness, i.e., individuals with large errors for elimination. Thus, individual neural networks with poor fit are discarded in the training process. Then, perform uniform mutation and arithmetic crossover operations on general individuals to obtain a new generation. After repeated training reaches the specified number of evolutions, the optimal model is obtained. Otherwise, it returns to the fitness calculation step to reiterate. The final individual is decoded to obtain all the connection weights in the neural network, which are used for actual prediction and quantitative evaluation indicators are obtained. Figure 8 is a detailed flowchart of the GA-BP HFMD prediction model.

## 5. Construction of HFMD Early-Warning Model Based on Big Data

The traditional HFMD epidemic warning model is mainly based on two major methods of time and space, namely the moving percentile prediction method and the spatial scanning statistical method. It also needs to use the repeated warning kicking algorithm to remove duplicates. The early-warning method is only based on historical data before and after the same period. It does not consider that the HFMD outbreak may be related to related weather and demographic factors. Blind warning and the warning process are cumbersome and wrong. There is a situation of blind warning every day and everywhere.

**Figure 9:**
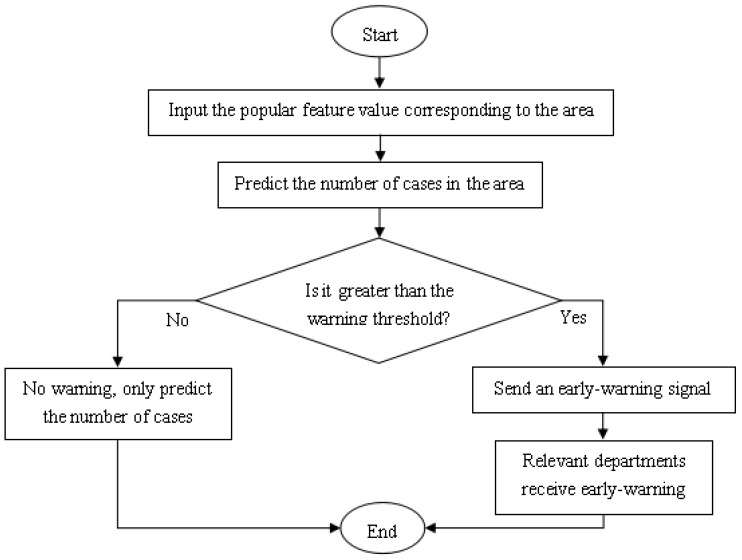
Flow chart of HFMD epidemic warning model.

As there is no yet a complete and accurate early-warning mechanism for infectious diseases, HFMD epidemic early-warning methods based on adjustable parameters, moving percentiles and a combination of the two are proposed, mainly proposed different methods for the setting of HFMD early-warning threshold. The HFMD epidemic early-warning model is mainly based on the number of HFMD cases in the region generated by the above HFMD epidemic prediction model and the number of cases in the same period in history. That is, whether there will be an outbreak of HFMD epidemic in the early warning area, Or according to historical data in the same period, relevant health departments need to be warned to increase the vigilance of the HFMD epidemic in the region. The overall process of the model is shown in Figure 9.

### 5.1. HFMD Epidemic Warning Model Based on Adjustable Parameters

The parameters are adjustable, referring to different HFMD epidemic areas, according to the epidemic time of different seasons, the corresponding characteristic parameters in the HFMD prediction model can be dynamically adjusted according to the actual local conditions. The corresponding features can be obtained by the feature selection method, and the number of patients output by the feature parameters through the model is used as the early-warning threshold of HFMD infectious diseases. If the actual predicted value exceeds the early-warning threshold, the system will issue an early-warning signal. For example, when the minimum temperature, air humidity, the number of illnesses last week, the number of children aged 0–6, and the number of weeks are 24 degrees Celsius, 40%, 45 cases, 28 thousand people, and 16:00, through the HFMD epidemic prediction model, the incidence under this feature is obtained and used as the early-warning threshold. These characteristic values can be dynamically adjusted according to different regions and different times.

### 5.2. HFMD Epidemic Warning Model Based on Historical Percentile Method

First, establish a database of local historical cases of HFMD with the city as the unit, refer to the historical incidence of the same period in the past 3–5 years and two cycles before and after the same period. Like the forecast period, the general historical period is seven days. Then get the percentile (usually 80% after sorting from small to large) from the historical incidence as the early-warning threshold. When the predicted incidence in the statistical period is greater than this early-warning threshold, the system will automatically send an early-warning signal to relevant departments in the observation area within one day. For example, it is predicted that the weekly incidence of the area will reach 100. Among the nine incidence data of the same period and before and after the past three years, the incidence at the 80th percentile is 80. Then the system will send an early-warning signal to the relevant departments in the forecast area to remind the area that there may be an outbreak, or the need to strengthen prevention and control higher than the historical level.

### 5.3. HFMD Epidemic Warning Model Based on Threshold Comparison

**Figure 10:**
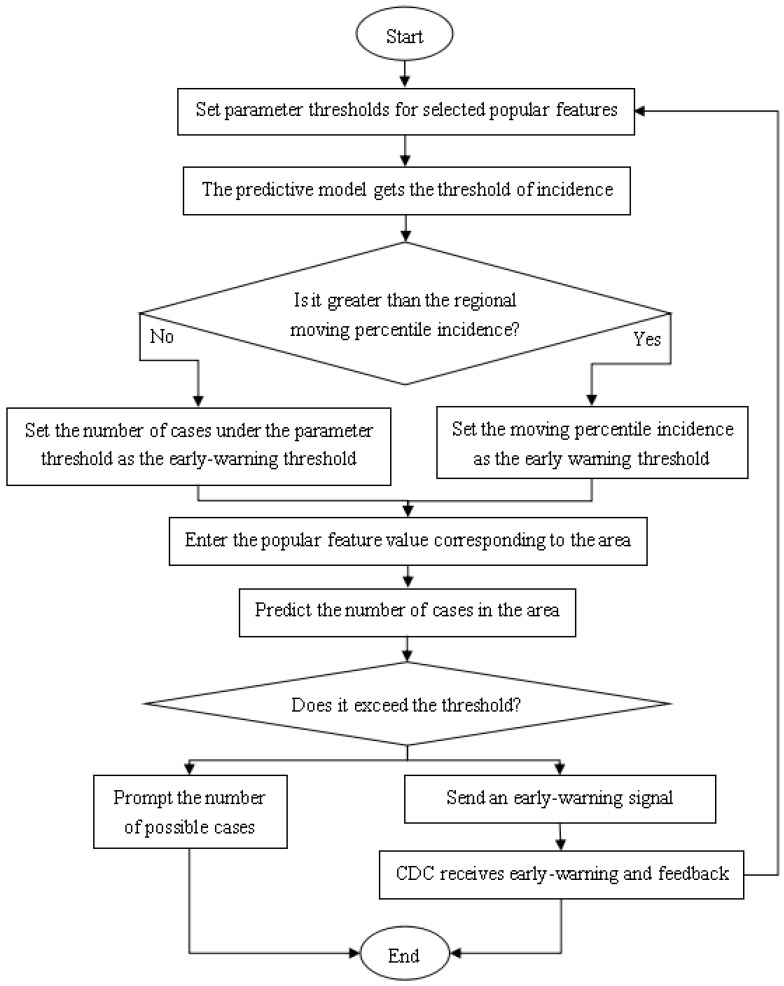
Flow chart of HFMD epidemic warning model based on threshold comparison.

Threshold comparison is to compare the two early-warning thresholds obtained by the above-mentioned parameter adjustable method and the historical percentile method, and use the smallest as the new early-warning threshold. The flow chart of the HFMD epidemic warning model based on threshold comparison is shown in Figure 10.

First, set the characteristic thresholds of the influencing factors of the HFMD epidemic, i.e., under the corresponding weather factors and demographic factors, the number of possible HFMD incidences is the number at risk of HFMD outbreaks, and these characteristic values are substituted into the HFMD prediction model to obtain the incidence number threshold.

Then, according to the local database of historical HFMD cases, calculate the 80th percentile number of cases in the same cycle and two swing cycles in the past three years, and use this value as another threshold for early-warning.This threshold is compared with the early-warning threshold obtained by parameter adjustment, and the minimum incidence threshold is used as the early-warning threshold of HFMD early-warning model.

Finally, the HFMD epidemic prediction model is used to predict the number of local cases. When the number of cases exceeds the early-warning threshold, the local health department will send an early-warning signal and take corresponding measures after verification. At the same time, it can feed back suggestions, continuously adjust the characteristic parameter thresholds, and improve the precise HFMD early-warning mechanism; When this incidence does not exceed the warning threshold, no warning signal is issued, only the number of HFMD infections that may occur in the local area.

## 6. Experiments

### 6.1. Predictive Model Evaluation Index

#### 6.1.1. Goodness of Fit

Goodness of fit refers to the degree of fit of the regression model to the observations, and its measurement statistic is the coefficient of determination $R^{2}$. The value range of $R^{2}$ is [0, 1]. The larger the value in this range, the better the fitting effect of the regression equation to the training sample; on the contrary, the worse the fitting degree. When the goodness of fit is negative, It shows that the fitting effect of this regression model is too poor and has no practical significance. Suppose *y* is the value to be fitted, its mean value is $\bar{y}$, the fitted predicted value is rounded to $\hat{y}$, the total square sum (*SST*) is $\sum_{i=1}^{n}{\left(y_{i}-\bar{y}\right)}^{2}$, the regression square sum (*SSR*) is $\sum_{i=1}^{n}{\left(\hat{y_{i}}-\bar{y}\right)}^{2}$, and the residual square sum (*SSE*) is $\sum_{i=1}^{n}{\left(y_{i}-\hat{y_{i}}\right)}^{2}$, then SST=SSR+SSE, the calculation method of the determination coefficient is as follows:

(2) $$R^{2}=\frac{SSR}{SST}=1-\frac{SSE}{SST}$$

Generalization ability is an important indicator for detecting regression prediction performance. Therefore, when designing a regression model, it is necessary to consider not only the model’s correct prediction of the required regression prediction object, but also the prediction effect of the model on the new data. The preprocessed data is divided into training data and test data in a ratio of 7:3. The evaluation index of the final model is the sum of the weights of the goodness of fit of the two, and the weight ratio is 3:7.

#### 6.1.2. Mean Absolute Error

The mean absolute error (MAE) refers to the average of the absolute value of the difference between multiple predicted values and the true value. In the HFMD epidemic prediction model, the average absolute error indicates the degree of deviation between the number of HFMD cases predicted by the model and the number of true cases when the number of HFMD cases is predicted in different regions or at different times. The smaller MAE, the more accurate the mode. The larger MAE, the worse the predictive ability of the model. Therefore, the magnitude of the average absolute error MAE reflects the pros and cons of the model, and its calculation formula is as follows:

(3) $$MAE=\frac{1}{n}\sum_{i=1}^{n}\left|y_{i}-y\right|$$

where, *n* represents the number of predictions, $y_{i}$ represents the predicted value, and *y* represents the true value.

#### 6.1.3. Accuracy within Error

Similar to the accuracy rate in the classification problem, in order to avoid the influence of interference factors, the accuracy rate within the error is introduced. The accuracy within error (AWE) refers to the ratio of the number of samples correctly fitted by the regression model obtained through training to the total number of training samples in the regression analysis process of predicting integer dependent variables within a certain error tolerance. To a certain extent, AWE explains the generalization ability of the regression model. The higher the AWE, the stronger the fitting ability of the model, and the lower AWE, indicating the weak fitting ability of the regression model, which ranges between 0 and 100%. The calculation formula of AWE is as follows:

(4) $$AWE=\frac{n}{N}\times 100\%\;\left(n:=n+1\;when\;\left|f\left(X_{i}\right)-y_{i}\right|\leq error_{people}\right)$$

where *n* represents the number of samples correctly predicted by the regression, and *N* represents the total number of samples. When the absolute value of the error between the predicted value and the true value is less than the specified error range, add 1 to the number of correctly fitted samples *n*, until all samples are trained, and the final number of samples correctly predicted is obtained.

When predicting the number of HFMD cases, the number of cases of the dependent variable is an integer number. Therefore, the predicted value must be rounded up, and then the predicted value after processing is compared with the true value. If the difference between the two is within the allowable range of the number of errors, it is considered that the trained model predicts the sample correctly, otherwise it is considered that there is a large error in the sample prediction. According to the analysis of infectious disease researchers and the regression model, the number of errors is five, i.e., when the difference between the predicted value and the true value does not exceed five, it is determined that the model fits this sample correctly.

### 6.2. Early-Warning Model Evaluation Index

#### 6.2.1. Warning Rate

The warning rate (WR) refers to the ratio of the number of samples that use the early warning model to send out early warning signals to the total number of samples, and its value is within the range of [0%, 100%]. Appropriate warning rate can reflect the difference of the model to different test data, and avoid the phenomenon of full warning and no warning. If the warning rate is too large or too small, it reflects the large error of the early warning model and the failure of correct warning.

#### 6.2.2. Accuracy Rate

Accuracy rate (ACR) refers to the ratio of the number of samples with the same early warning results of the model to the test data and the real early-warning results to the total sample, reflecting the accuracy of the HFMD epidemic early-warning model, and its value range is Between [0, 1].

The higher the accuracy rate, the better the prediction ability of the early-warning model, and the lower the accuracy rate, the worse the prediction ability of the early-warning model. Therefore, the accuracy rate can truly reflect the prediction effect of the HFMD epidemic early-warning model.

### 6.3. Comparison of Different Prediction Time and Space Accuracy

**Figure 11:**
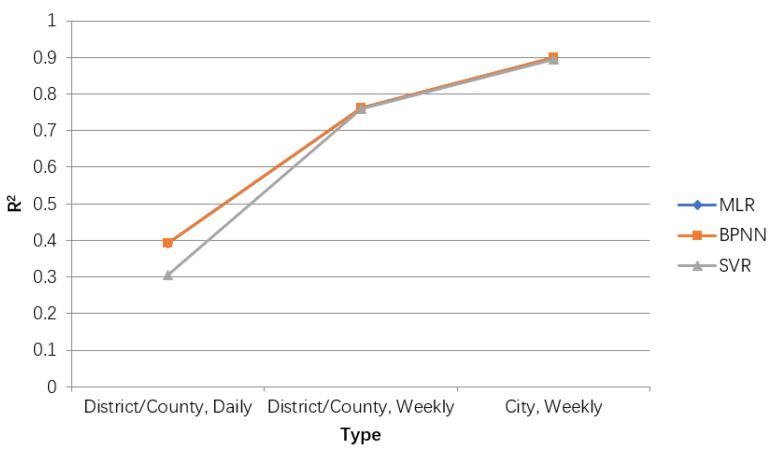
Comparison line chart of results of different prediction accuracy. The value represents results for R2, MAE/10, AWE for different parameters settings.

To obtain a more accurate prediction model of the number of HFMD cases, the different temporal and spatial precisions were compared. From the finest time accuracy (day) and spatial accuracy (districts and counties) to a week and prefectures, respectively, linear regression, BP neural network and SVR are used to fit predictions. Because the average and variance of the number of cases in different time and space accuracy are very different, only the goodness of fit $R^{2}$ is used to compare the models. The experimental results are shown in Table 2, and the broken line graph is shown in Figure 11. With the expansion of time and space accuracy, the goodness of fit of the model doubles. At the same time, in the comparison of three different methods, the BP neural network prediction model has the largest $R^{2}$, so the city-level weekly prediction model based on the BP neural network has the highest accuracy.

### 6.4. Comparative Analysis of Forecasting Models

**Figure 12:**
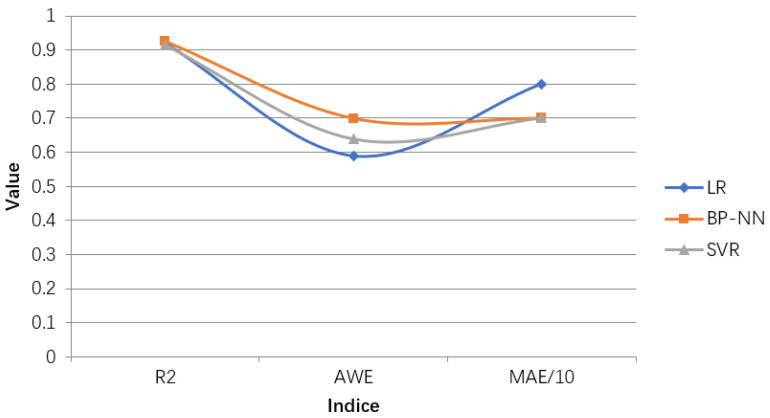
Line chart comparing the training results of different machine learning regression algorithms. The value represents results for R2, MAE/10, AWE for different parameters settings.

In this paper, by training and forecasting numerical variables, based on the weekly incidence of HFMD in the city and the weekly weather data that affects the epidemic and the child population data, the relevant variable analysis of the influencing factors is carried out. After that, a multivariate joint feature selection method based on correlation analysis was used to screen out a subset of features suitable for building a linear model, including the weekly ordinal number of the statistical time, the incidence of the previous week, the weekly average air pressure, and the number of children aged 0–6. At the same time, a subset of features suitable for establishing a nonlinear model is obtained, including the weekly ordinal number of the statistical time, the incidence of the previous week, the weekly average air pressure, the weekly minimum temperature, the weekly air humidity, the wind level and the number of children aged 0–6. Three regression methods were used to establish a model to fit the weekly incidence. Under different evaluation indicators, the training results of each model are shown in Table 3 and Figure 12. The analysis shows that the $R^{2}$ of the BP neural network reaches the maximum and the MAE reaches the minimum. At the same time, when the prediction error does not exceed the MAE, the AWE reaches its maximum value. Therefore, the HFMD epidemic prediction model based on BP neural network performs best, and the fitting effect is relatively best.

### 6.5. GA Tuning Parameter Analysis

When optimizing the connection weights of the BP neural network prediction model with a good fit effect, it is necessary to adjust and compare the hyperparameters such as the number of individuals in the population, the number of generations, the crossover and mutation probability in the genetic algorithm. Therefore, in this section, the hyperparameters in Table 4 are adjusted from the default values, and the optimal value is selected as the result of this test after three tests under the same conditions. It is used to evaluate the comparison results of $R^{2}$, MAE and AWE recording the changes of various hyperparameters, find the relevant hyperparameters of the model with the strongest generalization ability, and find the HFMD epidemic prediction model with the highest prediction accuracy based on these hyperparameters.

#### 6.5.1. Impact of the Number of Generations

**Figure 13:**
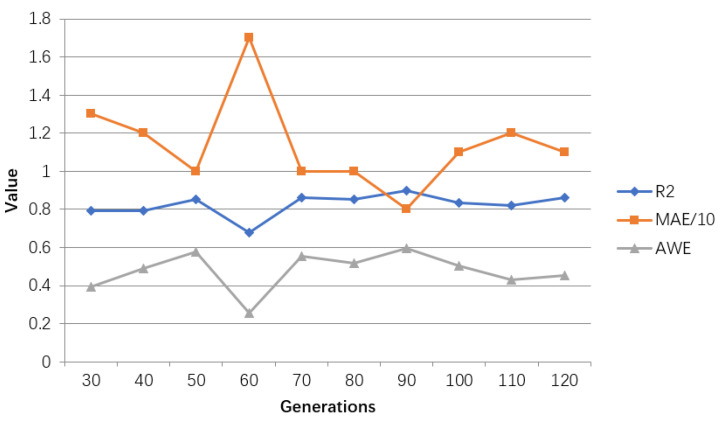
Line chart of adjustment results of hyperparameters in GA-BP model. The value represents results for R2, MAE/10, AWE for different parameters settings.

The model evaluation results of adjusting the number of generations are shown in Table 5, and the line graph shown in Figure 13 is drawn accordingly.

According to Table 5 and Figure 13, when the number of generations reaches 90 times, the goodness of fit and the accuracy within error achieve the maximum value. When it is greater than or less than this value, these two indicators will become smaller and affect the prediction effect. At the same time, MAE reaches the minimum value, and increases whenever it is greater or less than this value. Therefore, the population evolution is iterated 90 times, and the model is optimal.

#### 6.5.2. Effect of Population Size

**Figure 14:**
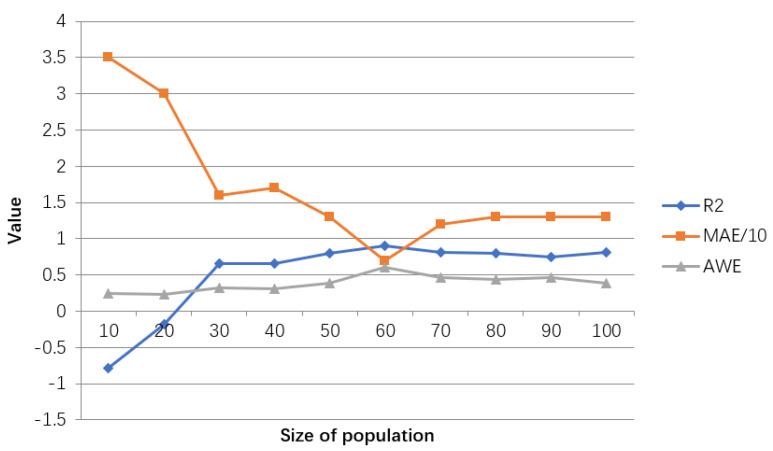
Line chart of adjustment results of hyperparameters in GA-BP model. The value represents results for R2, MAE/10, AWE for different parameters settings.

The evaluation results of the model for adjusting the population size are shown in Table 6, and the line graph shown in Figure 14 is drawn accordingly.

According to Table 6 and Figure 14, when the population size reaches 60, the goodness of fit and AWE take the maximum value. When it is greater than or less than 60, the value decreases. At the same time, MAE is the smallest, and when it is greater than or less than 60, its value will increase. Therefore, the model with a population size of 60 is optimal.

#### 6.5.3. Impact of Crossover Probability

**Figure 15:**
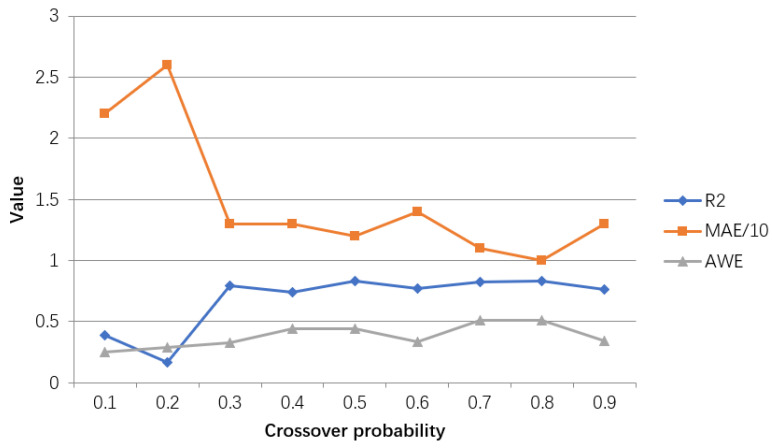
Line chart of the adjustment results of the crossover probability of the hyperparameters in the GA-BP model. The value represents results for R2, MAE/10, AWE for different parameters settings.

The model evaluation results for adjusting the cross probability are shown in Table 7, and the line graph shown in Figure 15 is drawn accordingly.

It can be obtained from Table 7 and Figure 15 that when the crossover probability reaches 0.8, the goodness of fit and AWE achieve the maximum value, while MAE is the smallest. When the crossover probability is not 0.8, the relevant index values are not ideal. Therefore, the GA model with a crossover probability of 0.8 performs best.

#### 6.5.4. Impact of Gene Mutation Probability

**Figure 16:**
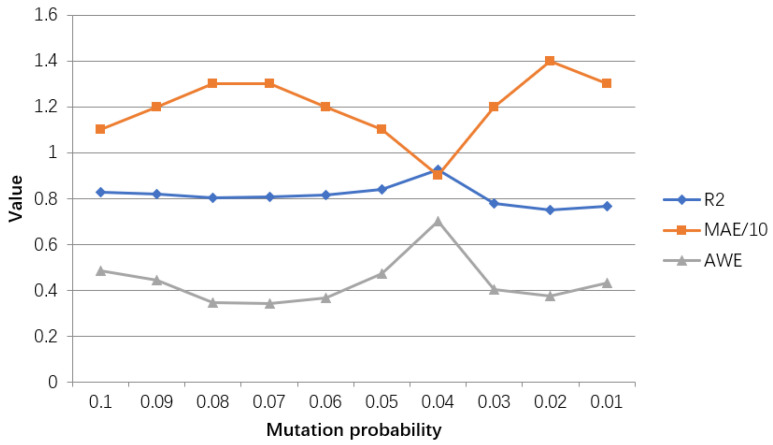
Line chart of adjustment results of hyperparameter mutation probability in GA-BP model. The value represents results for R2, MAE/10, AWE for different parameters settings.

The evaluation results of the model for regulating the probability of gene mutation are shown in Table 8, and the line graph shown in Figure 16 is drawn accordingly.

It can be obtained from Table 8 and Figure 16 that when the mutation probability is 0.04, the goodness of fit and AWE reach a good result, the maximum value is selected, and MAE is minimum. Higher or lower than this probability will make the relevant index value worse. Therefore, the GA model with a mutation probability of 0.04 has the best performance and a better effect.

Through the adjustment and comparison of these four hyperparameters in GA, it is found that the individual population is 60, the number of evolutionary iterations is 90, the gene crossover probability is 0.8, and the gene mutation probability is 0.04. At this time, the neural network HFMD prevalence prediction model based on GA has the strongest generalization ability, the smallest error, and best results.

### 6.6. Comparative Analysis of Early-Warning Models

According to our experiment, we have built the GA-BP HFMD prediction model. We set the critical eigenvalue of HFMD outbreak and substitute the eigenvalue into the prediction model to obtain the first HFMD outbreak threshold. Then, the samples of test data were input into three early warning models, and the 80 percentile incidence of the region was calculated as the second HFMD outbreak threshold for the same period of 3 years and 2 weeks before and after the same period. Finally, the WR and ACR values of different warning models were counted, as shown in Table 9.

According to the comparison, we could see that although warning model based on adjustable parameters has the best ACR, its WR is too small, so it is not generalized. Therefore, we conclude that the warning-model based on threshold comparison should be the optimal one.

## 7. Conclusions

This paper proposes a prediction and early-warning model for HFMD and the model uses big data. Data used in this paper are patient data and weather data. We can obtain a more accurate early-warning effect by constructing integrated prediction and early-warning model based on big data.

This paper constructs the prediction model by using GA to optimize the BP neural network. The best prediction accuracy could be gain as 92.56%. Then we explores the various construction methods of early-warning model. Through the comparison of experiment results, it is found that the early-warning model based on the comparison of threshold has the highest accuracy. And the optimal accuracy of the early-warning method is around 87.28%.There are still many parts that can be optimized in the research of this paper. For example, we would want to add more factors to enhance the accuracy of the prediction model. We will continue to study in depth accordingly.

## Figures and Tables

**Table 1:** HFMD epidemiological research data sheet.

Variable Name	Basic Meaning of Data	Type of Data	Range
weekofyear	week of statistical time	discrete variable	1–53
addID	statistical area code	discrete variable	1401–1411
count	number of infections	integer variable	0–
last_count	number of infections in the previous week	integer variable	0–
highTem	average maximum temperature	continuous variable	0–39
lowTem	average minimum temperature	continuous variable	0–39
windLevel	average wind level	continuous variable	0–12
p_highTem	average maximum temperature before the incubation period	continuous variable	0–39
p_lowTem	average minimum temperature before incubation period	continuous variable	0–39
p_windLevel	average wind level before the incubation period	continuous variable	0–12
hTemDiff	maximum temperature difference	continuous variable	−15–15
lTemDiff	lowest temperature difference	continuous variable	−15–15
windLevDiff	wind power difference	continuous variable	−3–3
wet	average air humidity	continuous variable	0–100
sunshine	average sunshine duration	continuous variable	0–10
pressure	average air pressure	continuous variable	−2–2
p_wet	average air humidity before incubation period	continuous variable	0–100
p_sunshine	average sunshine duration before incubation period	continuous variable	0–10
p_pressure	average pressure before the incubation period	continuous variable	−2–2
wetDiff	average humidity difference	continuous variable	−100–100
sunDiff	average sunshine duration difference	continuous variable	−10–10
pressDiff	average air pressure difference	continuous variable	−5–5
Children	number of children under 6	integer variable	1–

**Table 2:** Experimental results of different time precision and spatial precision prediction.

Forecast Model Name	District/County, Daily Forecast	District/County, Weekly Forecast	City-Level, Weekly Forecast
LR	0.3914	0.7611	0.8983
BP-NN	0.3931	0.7613	0.8994
SVR	0.3041	0.7597	0.8948

**Table 3:** Training results of different machine learning regression algorithms.

Evaluation Index	LR	BP-NN	SVR
$R^{2}$	0.9193	0.9243	0.9142
MAE	8	7	7
AWE	58.94%	69.94%	63.85%

**Table 4:** Hyperparameters related to genetic algorithm in GA-BP neural network model.

Hyperparameter Name	Description	Default
Population size	number of individuals in the population	70
Number of generations	population evolution times	60
Mutation probability	probability of genetic mutation on individual chromosomes	0.1
Crossover probability	probability of genetic recombination on chromosomes of two individuals	0.9

**Table 5:** Evaluation table for adjusting the number of generations of hyperparameters in GA-BP model.

Number of Generations	$R^{2}$	MAE	AWE
30	0.7933	13	0.3922
40	0.7916	12	0.4902
50	0.8538	10	0.5752
60	0.6798	17	0.2549
70	0.8622	10	0.5556
80	0.8539	10	0.5163
90	0.8992	8	0.5962
100	0.8328	11	0.5033
110	0.8209	12	0.4314
120	0.8628	11	0.4510

**Table 6:** The population size adjustment evaluation table of the hyperparameters in the GA-BP model.

Population Size	$R^{2}$	MAE	AWE
10	−0.7920	35	0.2418
20	−0.1791	30	0.2353
30	0.6508	16	0.3203
40	0.6592	17	0.3072
50	0.7964	13	0.3856
60	0.9023	7	0.6053
70	0.8131	12	0.4575
80	0.7942	13	0.4379
90	0.7422	13	0.4575
100	0.8052	13	0.3856

**Table 7:** Adjustment evaluation table of cross probability of hyperparameters in GA-BP model.

Crossover Probability	$R^{2}$	MAE	AWE
0.1	0.3900	22	0.2484
0.2	0.1680	26	0.2876
0.3	0.7922	13	0.3268
0.4	0.7411	13	0.4444
0.5	0.8301	12	0.4444
0.6	0.7688	14	0.3333
0.7	0.8258	11	0.5074
0.8	0.8341	10	0.5098
0.9	0.7635	13	0.3399

**Table 8:** Adjustment evaluation table of hyperparameter mutation probability in GA-BP model.

Mutation Probability	$R^{2}$	MAE	AWE
0.10	0.8270	11	0.4837
0.09	0.8194	12	0.4444
0.08	0.8036	13	0.3464
0.07	0.8053	13	0.3399
0.06	0.8137	12	0.3660
0.05	0.8400	11	0.4706
0.04	0.9256	9	0.7011
0.03	0.7796	12	0.4033
0.02	0.7484	14	0.3725
0.01	0.7643	13	0.4314

**Table 9:** Comparison of different early-warning models.

	Adjustable Parameters	Historical Percentile	Threshold Comparison
WR	4.13%	29.08%	32.02%
ACR	98.62%	85.46%	87.28%

## Data Availability

Not applicable.
